# Real-world use patterns, effectiveness, and tolerability of sacituzumab govitecan for second-line and later-line treatment of metastatic triple-negative breast cancer in the United States

**DOI:** 10.1007/s10549-024-07412-9

**Published:** 2024-06-21

**Authors:** Kevin Kalinsky, Laura Spring, Clinton Yam, Manali Ajay Bhave, Ioanna Ntalla, Catherine Lai, Nikoleta Sjekloca, Brian Stwalley, Michael Stokes, Aliki Taylor, Rita Nanda

**Affiliations:** 1 Department of Medical Oncology, Winship Cancer Institute, Emory University, 1365 Clifton Road Northeast, Bldg C, Fl 2, Atlanta, GA 30322 USA; 2 Division of Hematology/Oncology, Massachusetts General Hospital Cancer Center and Harvard Medical School, Boston, MA USA; 3 Department of Breast Medical Oncology, The University of Texas MD Anderson Cancer Center, Houston, TX USA; 4 Global Medical Affairs, Gilead Sciences Europe Ltd., Stockley Park, UK; 5 US Medical Affairs, Gilead Sciences, Inc., Foster City, CA USA; 6 Data Analytics, Evidera, Bethesda, MD USA; 7 Department of Medicine, The University of Chicago Medicine, Chicago, IL USA; 8 Real-World Evidence, Gilead Sciences Europe Ltd., Stockley Park, UK; 9 Clinical Development, Gilead Sciences, Inc., Foster City, CA, USA; 10 Breast Oncology Program, Winship Cancer Institute, Emory University, Atlanta, GA, USA

**Keywords:** Sacituzumab govitecan, Triple-negative breast cancer, Real-world clinical outcomes, Second-line, Metastatic, Treatment patterns

## Abstract

**Purpose:**

Patients with metastatic triple-negative breast cancer (mTNBC) have poor prognosis and limited treatment options. Sacituzumab govitecan (SG), a Trop-2–directed antibody–drug conjugate, is approved for patients with mTNBC who have received ≥ 2 systemic therapies (≥ 1 in the metastatic setting) based on the ASCENT study (NCT02574455). The current study describes real-world SG use and outcomes in patients with mTNBC in the United States.

**Methods:**

This retrospective, observational study included adult patients with mTNBC from the ConcertAI Patient360™ database who received SG in the second line (2L) and later from April 2020 to May 2022. SG use patterns, effectiveness, and tolerability are described.

**Results:**

This analysis included 230 patients (median age 60 years, 26% Black, 17% with ECOG performance status ≥ 2, 66% in community settings; median of 2 prior lines of treatment in the metastatic setting); median follow-up was 7.2 months. Median (95% CI) real-world overall survival was 10.0 (8.3–11.1) months for all patients and 13.9 (9.8-not estimable) months in the 2L subgroup (n = 77). Granulocyte-colony stimulating factor (G-CSF) was administered concomitantly with SG in 134 (58%) patients; 35 (15%) received G-CSF for the first time. Median (IQR) time from SG start to G-CSF use was 8.5 (8.0–29.0) days. Seventeen (7%) patients discontinued SG due to toxicity.

**Conclusions:**

Using a real-world, ethnically diverse population of patients with mTNBC presenting with poor prognosis, these data reinforced the findings from ASCENT. In routine clinical practice, SG is an effective treatment in the 2L setting, consistent with treatment guidelines.

**Supplementary Information:**

The online version contains supplementary material available at 10.1007/s10549-024-07412-9.

## Introduction

Triple-negative breast cancer (TNBC) is an aggressive form of breast cancer (BC) accounting for approximately 10–15% of BC cases in the United States [1, 2]. It is defined by the American Society of Clinical Oncology and the College of American Pathologists (ASCO/CAP) as estrogen/progesterone receptor expression < 1% and human epidermal growth factor receptor 2 (HER2) immunohistochemistry (IHC) 0, 1 + , or IHC 2 + /in situ hybridization-negative [3, 4]. TNBC is associated with moderate/high-grade large tumors, visceral metastases, high rates of distant recurrence, and poor prognosis [5–8]. In the United States, the 5-year survival rate for all patients with TNBC is approximately 78%, decreasing to about 13% for patients with distant metastases [1]. Historically, the poor outcome for this subtype has largely been due to a lack of targeted treatments; however, this is improving with the development of novel therapies.

Sacituzumab govitecan (SG) is a Trop-2–directed antibody–drug conjugate that delivers SN-38 (the active metabolite of the topoisomerase inhibitor irinotecan) to tumor cells via internalization and to the surrounding tumor microenvironment via the bystander effect [9]. The pivotal phase III ASCENT study (NCT02574455) showed superior efficacy with SG compared with single-agent chemotherapy in 468 patients with metastatic TNBC (mTNBC). Median progression-free survival (PFS) was significantly longer with SG (5.6 vs. 1.7 months, respectively; hazard ratio [HR] for disease progression or death, 0.41; 95% confidence interval [CI] 0.32–0.52; *P* < 0.001). Median overall survival (OS) was also significantly longer (12.1 vs. 6.7 months, respectively; HR, 0.48; 95% CI 0.38–0.59; *P* < 0.001). These benefits were evident across all subgroups, including older and pretreated patients, patients with liver metastases, and patients who received prior immune checkpoint inhibitor therapy. SG was associated with a low incidence of adverse event (AE)-related discontinuations, and clinically relevant AEs—notably neutropenia and diarrhea—could be managed with established guidance [9].

SG was granted accelerated approval by the United States Food and Drug Administration in April 2020 based on the results from the phase I/II basket trial IMMU-132-01 (NCT01631552); full approval was granted in April 2021, based on the ASCENT trial, for the treatment of patients with unresectable locally advanced or mTNBC who have received two or more prior systemic therapies, at least one for metastatic disease, and is now available in multiple countries for this indication [10, 11].

Currently, treatment guidelines recommend chemotherapy as first-line therapy in mTNBC (± programmed cell death (ligand)-1 [PD-(L)1] targeted therapy, depending on ligand expression) and recommend SG as second-line (2L) therapy for patients with mTNBC who have received at least two prior therapies (at least one in the metastatic setting); SG may also be considered for later lines if not used as 2L therapy [12–14].

It is important to determine how SG is being utilized in real-world settings and to identify optimal treatment approaches for health care professionals. Due to the recent approval, there are few studies of SG use in patients with mTNBC in the real-world setting [15–20]. The objective of this study was to describe patient characteristics, treatment patterns, tolerability, and clinical outcomes in patients with mTNBC who received at least one dose of SG in the 2L and later-line setting in clinical practices in the United States.

## Methods

### Data sources and study design

This was a retrospective, observational cohort study (Supplementary Fig. 1). De-identified electronic health records were obtained from the ConcertAI Patient360™ database, an extensive clinical database that collates patient data from more than 400 academic and community oncology practices in the United States, including structured and unstructured records. Additional custom abstraction from physicians’ notes was applied to increase the sample size and the variables captured including dose modifications and reasons, treatment discontinuations and reasons, and real-world AEs (rwAEs) of interest (neutropenia, diarrhea, alopecia, and fatigue).

Eligible patients were aged ≥ 18 years with an mTNBC diagnosis and were required to have 2L and later line of SG treatment initiated between April 1, 2020, and May 31, 2022. The first SG administration that occurred on or after the metastatic diagnosis date was defined as the index (treatment start) date. The follow-up period included the time from the index date until death, last activity, or study end (August 31, 2022), whichever occurred first. Patients were excluded if they had any other primary cancer diagnosis within 5 years prior to treatment start (excluding non-metastatic, non-melanoma skin cancers).

Patient demographics and disease characteristics were described using values closest to the index date.

### Treatment patterns

SG treatment patterns were summarized, including line of therapy use in the metastatic setting, dosing patterns (including number of doses and starting dose), and duration of treatment. The start of therapy and advancement to next line of therapy was defined based on a regimen-based framework, where a change of regimen, rather than disease progression, was considered advancement to the next line. Switches from carboplatin to cisplatin, docetaxel to paclitaxel, capecitabine to 5-fluorouracil, and leucovorin to levoleucovorin were excluded from this framework.

### Effectiveness

Real-world OS (rwOS) was defined as the time (months) from the index date (first SG administration) to death from any cause and was determined for the overall population and for patients who received SG in 2L of therapy and in third and later line (3L +) of therapy. Time-to-next-treatment or death (TTNTD) was defined as the time (months) from the index date until start of next line of therapy or death, whichever came first; advancement to next line of therapy was defined based on the framework described above. Real-world PFS (rwPFS) was defined as the time (months) from the index date until disease progression or death, whichever came first; progression events, and corresponding dates, were curated from provider notes in the ConcertAI Patient360™ database of electronic health records. Time to SG discontinuation was defined as the time (months) from index date to SG treatment discontinuation or death, whichever came first. A sensitivity analysis was conducted to determine rwOS for patients with an index date prior to February 28, 2022, to allow for data accrual of 6 months before the end of study.

### Tolerability

rwAEs of clinical interest that occurred during SG treatment were recorded, specifically neutropenia, diarrhea, fatigue, and alopecia. Events that occurred during SG treatment were only considered to be treatment-related if there was no evidence of their occurrence during the 30-day period prior to the index date. rwAEs were described using abstracted data from patients’ electronic health records and did not include grade; thus, rwAEs of any grade are reported here. Laboratory data (absolute neutrophil count < 1500/mm^3^) and International Classification of Disease (ICD) diagnosis codes (Tenth Revision: ICD-10 D70 or Ninth Revision: ICD-9 288) were also used to identify patients with neutropenia [21]. Concomitant granulocyte-colony stimulating factor (G-CSF) utilization during SG treatment was assessed and included number of patients receiving G-CSF treatment (refers to the use as primary or secondary prophylaxis, or therapeutic use of short- or long-acting G-CSF), time from index date to first G-CSF administration, and if patients had received G-CSF with prior treatments. Duration of SG treatment by concomitant G-CSF use was also assessed. Treatment discontinuations and SG dose modifications (i.e., reduction or interruption), and reasons for them, were summarized using abstracted data from physicians’ notes. As categories were not mutually exclusive, some patients could have been counted in more than one category.

### Statistical analysis

All patients who met the eligibility criteria were included in data analyses. Data were summarized using descriptive statistics. Continuous data were summarized as mean (standard deviation [SD]) or median (interquartile range [IQR]), and categorical variables were described as number and proportions (95% CI). There was no imputation for missing variables.

Duration of SG treatment, in months, was calculated as (SG treatment end date—[SG treatment start date + 1])/30.4.

Median time-to-event analyses were performed for rwOS, TTNTD, rwPFS, and time to SG discontinuation. These outcomes were described using Kaplan–Meier analyses. For rwOS analyses, patients who were alive at the end of study or lost to follow-up were censored at their last confirmed activity date. For TTNTD analyses, patients who discontinued treatment without initiating another line of therapy, remained on SG treatment at study end, or were lost to follow-up were censored at these time points or at study end, whichever occurred first. For rwPFS analyses, patients who were still on SG treatment or had discontinued without progression, or were still alive at study end, or lost to follow-up, were censored at the last confirmed activity date or study end, whichever occurred first.

Stratified analyses of outcomes were performed for subgroups of interest, including concomitant G-CSF use (yes/no) and race (White, Black, Asian, and other/unknown). For the subset of patients from the sensitivity analysis (≥ 6 months follow-up) who had recurrent disease and who were treated with systemic anticancer therapy in the curative setting, and who initiated SG in 2L in the metastatic setting, rwOS was estimated by treatment-free interval duration (TFI; < 12 months/ ≥ 12 months). TFI was defined as duration from the end date of the last systemic anticancer therapy in the (neo)adjuvant setting to the date of mTNBC diagnosis.

## Results

### Patients

**Fig. 1:**
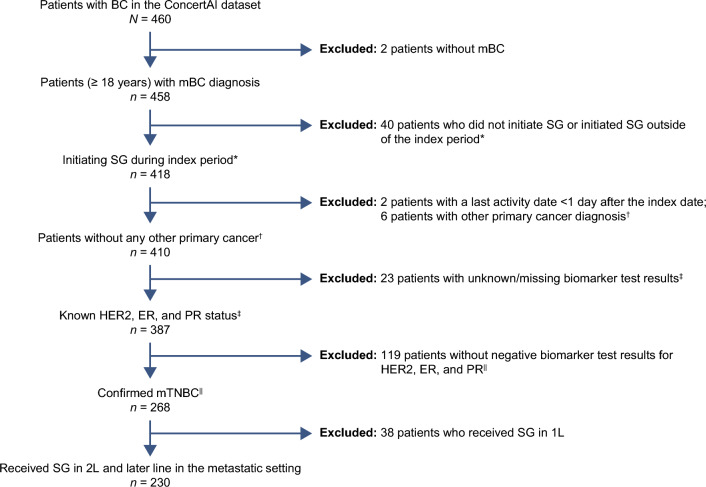
Patient selection. *1L*, first-line, *2L* second-line, *BC* breast cancer, *ER* estrogen receptor, *HER2* human epidermal growth factor receptor 2, *mBC* metastatic breast cancer, *mTNBC* metastatic triple-negative breast cancer, *PR* progesterone receptor, *SG* sacituzumab govitecan. *Within 30 days prior to, on, or after the first mBC date and during the index period. ^†^Up to 5 years prior to index date (except for non-metastatic, non-melanoma skin cancer). ^‡^Between 30 days prior to the first ever BC diagnosis date and ≤ 90 days after the index date. ^**||**^Biomarker test results closest to the index date were prioritized

Of the 460 patients in the ConcertAI Patient 360™ Breast Cancer custom dataset, 230 (50%) patients with mTNBC treated with SG in 2L and later line were selected based on the inclusion/exclusion criteria (Fig. 1).

The median (IQR) age was 60 (49–69) years; 146 (63%) patients were White and 59 (26%) were Black, and 40 (17%) patients had an Eastern Cooperative Oncology Group (ECOG) performance status ≥ 2 (Table 1).

**Table 1:** Baseline demographics and clinical characteristics for patients with mTNBC who received 2L and later-line SG treatment

Characteristics	Patient population (*N* = 230)
Age, median (IQR), y	60 (49–69)
< 65, No. (%)	139 (60)
≥ 65, No. (%)	91 (40)
Sex, No. (%)	
Female	230 (100)
Race, No. (%)	
White	146 (63)
Black	59 (26)
Asian	9 (4)
Other/Unknown	16 (7)
Ethnicity, No. (%)	
Hispanic or Latino	12 (5)
Other/Unknown	218 (95)
Treatment provider type, No. (%)	
Community	152 (66)
Academic	63 (27)
Unknown	15 (7)
Practice region, No. (%)	
Northeast	36 (16)
South	111 (48)
Midwest	50 (22)
West	33 (14)
BMI at index,^a^ median (IQR), kg/m^2^	26 (22–32)
ECOG performance status,^b^ No. (%)	
0 or 1	162 (70)
≥ 2	40 (17)
Unknown	28 (12)
Disease type, No. (%)	
Recurrent disease	170 (74)
De novo metastatic disease	41 (18)
Unknown	19 (8)
Time from mTNBC diagnosis to index date, median (IQR), months	11.8 (7.6–19.2)
Brain metastases at baseline, No (%)	17 (7)
Visceral metastases at baseline, No. (%)	167 (73)
PD-L1 expression status, No. (%)	
PD-L1 positive	22 (10)
PD-L1 negative	48 (21)
Unknown	160 (70)
*BRCA*1/2 mutation status, No. (%)	
Mutant	31 (13)
Wildtype	108 (47)
Unknown	91 (40)
TFI, No. (%)^c^	
< 12 months	57 (56)
≥ 12 months	44 (44)

*2L* second-line, *BMI* body mass index, *BRCA1/2* BReast CAncer gene 1/2, *ECOG* Eastern Cooperative Oncology Group, *IQR* interquartile range, *mTNBC* metastatic triple-negative breast cancer, *PD-L1* programmed cell death-ligand 1, *SG* sacituzumab govitecan, *TFI* treatment-free interval

^a^Based on 172 patients with data from 6 months before index date until 2 months after index date; value closest to and before or on the index date was used

^b^ECOG performance status records during the 6 month period before the index date until 2 months after the index date only were used. If ECOG performance status was not available during the 6 month period prior to the index date, Karnofsky scores were used instead (ECOG performance status 0 or 1 corresponds to Karnofsky scores 70–100 and ECOG performance status ≥ 2 to corresponds to Karnofsky scores ≤ 60)

^c^Based on 101 patients with recurrent disease and known TFI (measured from the end date of the last systemic anticancer therapy in the (neo)adjuvant setting to the date of mTNBC diagnosis)

Overall, 152 (66%) patients were treated in the community setting and 63 (27%) in the academic setting. In total, 41 (18%) patients were originally diagnosed with de novo mTNBC, 167 (73%) had visceral metastases, and 17 (7%) had brain metastases. The median (IQR) time from mTNBC diagnosis to index date or enrollment was 11.8 (7.6–19.2) months (Table 1). In the metastatic setting, patients received a median (IQR) of 2 (1–3) prior anticancer regimens (Table 2). Fifteen (7%) and 111 (48%) patients had previously been treated with a poly (adenosine diphosphate-ribose) polymerase inhibitor and a PD-(L)1 agent, respectively. Among all patients, 101 (44%) had received prior (neo)adjuvant therapy; 69 patients with recurrent disease had no evidence of receiving treatment in the curative setting.

**Table 2:** Prior therapies received in the metastatic setting for patients with mTNBC who received 2L and later-line SG treatment

Prior therapies	Metastatic setting (*N* = 230)	Metastatic & curative setting (*N* = 230)^a^
Previous anticancer regimens, median (IQR)	2 (1–3)	4 (2–5)
Previous chemotherapy drugs,^b^ No. (%)		
Taxanes	149 (65)	185 (80)
Carboplatin	96 (42)	109 (47)
Capecitabine	94 (41)	121 (53)
Anthracyclines	26 (11)	97 (42)
Cyclophosphamide	16 (7)	99 (43)
Previous use of PARPis,^c^ No. (%)	15 (7)	16 (7)
Previous use of PD-(L)1 inhibitors,^d^ No. (%)	111 (48)	111 (48)

*2L* second-line, *IQR* interquartile range, *mTNBC* metastatic triple-negative breast cancer, *PARPis* poly(adenosine diphosphate-ribose) polymerase inhibitors, *PD-(L)1* programmed cell death protein-(ligand) 1, *SG* sacituzumab govitecan

^a^101/230 (44%) patients had received prior (neo)adjuvant anticancer therapy

^b^The proportion of patients may add up to greater than 100% as the subgroups are not mutually exclusive

^c^PARPi included olaparib, talazoparib

^d^PD-(L)1 inhibitors included atezolizumab, pembrolizumab

Baseline demographics and clinical characteristics for patients who received SG in the 2L or in 3L + are reported in Supplementary Table 1.

### Sacituzumab govitecan treatment patterns

Of the 230 patients in this analysis, 77 (33%) received SG in 2L and 153 (67%) in 3L + ; 64 (28%), 43 (19%), and 46 (20%) patients received SG in 3L, 4L, and later line, respectively. Patients received a median (IQR) of 9 (5–16) SG doses. The maximum number of doses administered during the study period was 68. For the 170 patients with data on drug amount administered and weight at baseline, the median (IQR) starting dose was 10.0 (9.8–10.1) mg/kg. The median (IQR) SG treatment duration was 3.8 (2.1–7.0) months with maximum duration of 25.8 months during the study period. Among patients treated in 2L, median (IQR) treatment duration was 4.2 (2.1–8.0) months. Median (95% CI) time to SG treatment discontinuation was 3.0 (2.4–3.7) months. At the end of the study period, 124 (54%) patients had died; 21 (9%) patients were still on treatment.

### Effectiveness

**Fig. 2:**
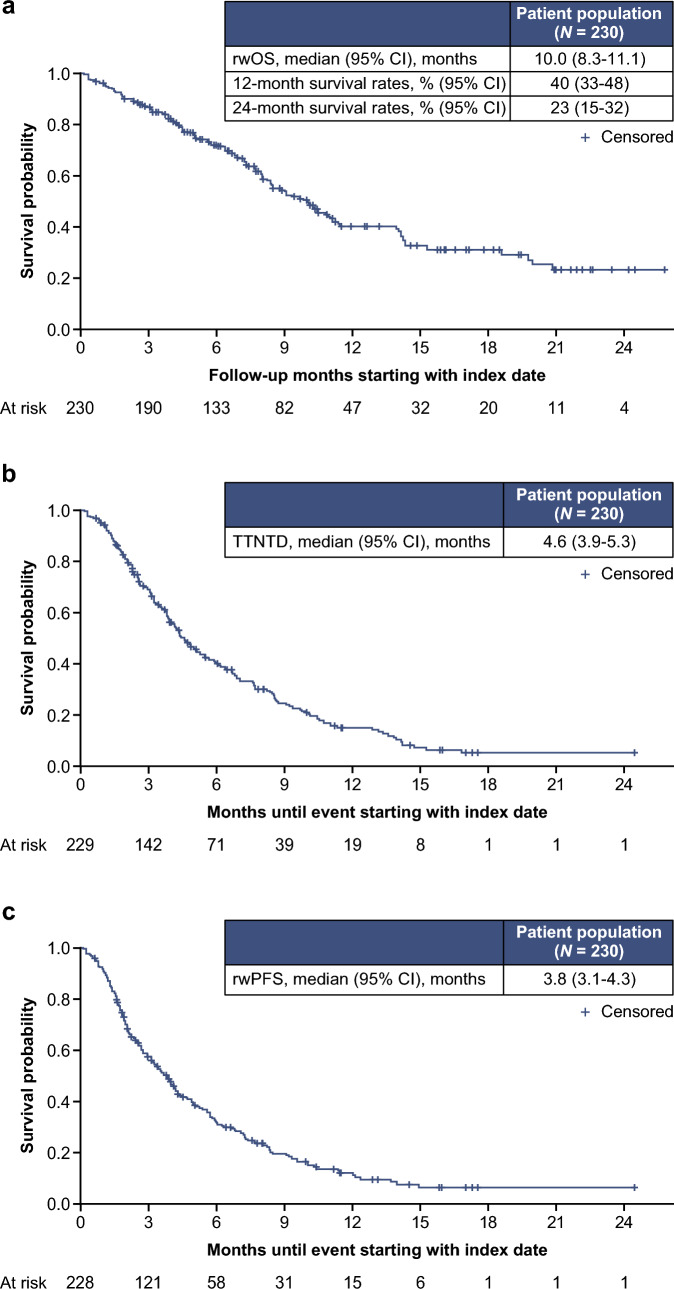
rwOS (**a**), TTNTD (**b**), and rwPFS (**c**) in patients with mTNBC who received 2L and laterline SG treatment. *2L* second-line, *CI* confidence interval, *mTNBC* metastatic triple-negative breast cancer, *rwOS* real-world overall survival, *rwPFS* real-world progression-free survival, *SG* sacituzumab govitecan, *TTNTD* time to next treatment or death

**Fig. 3:**
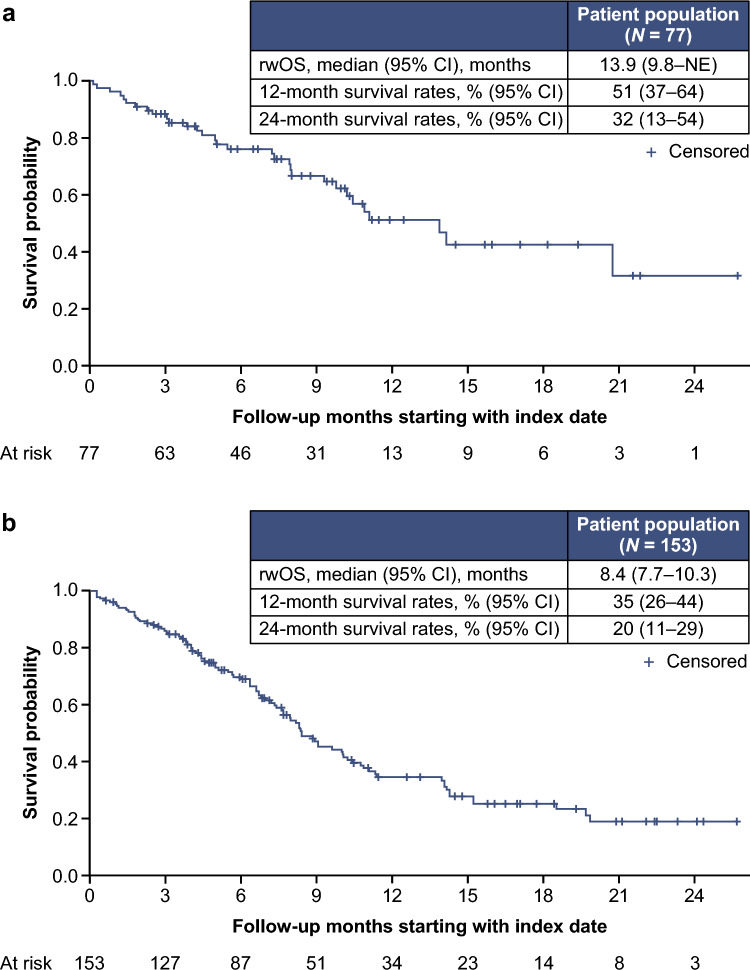
rwOS in patients with mTNBC who received SG in 2L (**a**) or 3L + (**b**). *2L* second-line, *3L* + third-line and later line, *CI* confidence interval, *mTNBC* metastatic triple-negative breast cancer, *NE* not estimable, *rwOS* real-world overall survival, *SG* sacituzumab govitecan

After a median follow-up of 7.2 months, the median (95% CI) rwOS was 10.0 (8.3–11.1) months among all patients (Fig. 2a). The 12 and 24 month survival rates (95% CI) for all patients were 40% (33–48%) and 23% (15–32%), respectively. In stratified analysis by SG line of therapy, rwOS was 13.9 (9.8–not estimable [NE]) months for patients receiving 2L SG and 8.4 (7.7–10.3) months for patients receiving 3L + SG (Fig. 3). The 12 and 24 month survival rates (95% CI) for patients receiving SG in 2L were 51% (37–64%) and 32% (13–54%), respectively. Median (95% CI) TTNTD was 4.6 (3.9–5.3) months (Fig. 2b; 2L: 4.8 [3.2–6.9] months; 3L + : 4.4 [3.8–5.5] months); rwPFS was 3.8 (3.1–4.3) months (Fig. 2c; 2L: 4.9 [2.9–6.0] months; 3L + : 3.5 [2.7–4.2] months).

For the 209 patients included in the sensitivity analysis (allowing for 6 month data accrual), median (95% CI) rwOS was 9.8 (8.2–10.9) months (2L: 13.9 [9.8–NE] months, *n* = 69; 3L + : 8.4 [7.3–10.1] months, *n* = 140).

### Tolerability

Fatigue was reported in 104 (45%) patients, neutropenia in 77 (33%), diarrhea in 70 (30%), and alopecia in 26 (11%).

G-CSF was administered concomitantly with SG in 134 (58%) patients. Most of these patients (99/134 [74%]) had also received G-CSF with previous anticancer treatments. Among all treated patients, 35 (15%) received G-CSF for the first time during treatment with SG. For the 35 patients initiating G-CSF for the first time during SG treatment, 74% received G-CSF in their first SG cycle with median time from SG start to G-CSF administration being 8.5 (IQR 8.0–29.0) days. The earliest administration of G-CSF occurred 2 days after the start of SG treatment and the latest occurred after 92 days post-treatment. Among the 96 patients who did not receive G-CSF while on SG treatment, 54 (56%) had received G-CSF with previous anticancer treatments. Furthermore, among the patients with concomitant G-CSF use (*n* = 134), the median (IQR) SG treatment duration was 4.0 (2.2–7.5) months and the maximum number of doses administered was 68. Among the patients who did not receive G-CSF during SG treatment (*n* = 96), the median (IQR) treatment duration was 3.3 (1.4–6.3) months and the maximum number of doses administered was 54.

Among all patients, 79 (34%) had SG dose reductions and 133 (58%) had SG dose interruptions documented in their records. Toxicity was the reason for dose reduction or interruption in 26% and 39% of patients, respectively. In total, 209/230 (91%) patients discontinued SG treatment; 128 (57%) due to disease progression, 17 (7%) due to toxicity, 11 (5%) due to death, 52 (23%) due to other reasons, and 3 (1%) due to unknown reasons (categories were not mutually exclusive).

### Stratified analyses

Median (95% CI) rwOS was similar for patients who did (*n* = 134) or did not (*n* = 96) receive G-CSF concomitantly with SG (9.1 [7.7–11.4] and 10.2 [8.3–14.2], respectively) and these data were consistent with the overall population. Similarly, median (95% CI) rwOS did not vary across racial subgroups (White [*n* = 146]: 9.1 [8.0–11.1]; Black [*n* = 59]: 10.1 [7.7–18.6]; Asian [*n* = 9]: 8.4 [3.8–NE]) and these findings were in line with the results in the overall population. Overall, similar trends were observed for TTNTD and rwPFS (Table 3).

**Table 3:** Stratified analyses of clinical outcomes by subgroups in patients with mTNBC who received 2L and later-line SG treatment

	*N*	rwOS, median (95% CI), months	TTNTD, median (95% CI), months	rwPFS, median (95% CI), months
		All patients (*N* = 230)		
Concomitant use of G-CSF				
Yes	134	9.1 (7.7–11.4)	4.7 (3.9–5.7)	4.1 (3.4–5.0)
No	96	10.2 (8.3–14.2)	4.4 (3.2–6.0)	3.2 (2.4–4.3)
Race				
White	146	9.1 (8.0–11.1)	4.6 (3.9–5.9)	4.1 (3.1–4.9)
Black	59	10.1 (7.7–18.6)	4.0 (3.1–7.6)	3.2 (2.3–5.0)
Asian	9	8.4 (3.8–NE)	4.2 (3.2–NE)	3.8 (2.3–5.0)
Other/unknown	16	14.0 (5.7–NE)	5.5 (2.3–13.8)	3.5 (0.9–7.7)
TFI^a^				
< 12 months	18	11.1 (8.0-NE)	4.0 (1.5–7.6)	4.6 (1.6–5.7)
≥ 12 months	16	10.9 (5.0–NE)	3.1 (1.4–5.0)	2.8 (1.6–4.0)

*2L* second-line, *CI* confidence interval, *G-CSF* granulocyte colony stimulating factor, *mTNBC* metastatic triple-negative breast cancer, *NE* not estimable, *rwOS* real-world overall survival, *rwPFS* real-world progression-free survival, *SG* sacituzumab govitecan, *TFI* treatment-free interval, *TTNTD* time to next treatment or death

Due to rounding, percentages may not add up to 100%

^a^Of 101 patients with recurrent disease and known TFI (time from end date of last systemic anticancer therapy in the (neo)adjuvant setting to date of mTNBC diagnosis), 34 patients received SG in 2L in the metastatic setting no later than February 2022

Among the 230 patients in the overall population, 77 had recurrent disease and had received systemic therapy in the (neo)adjuvant setting before receiving SG as 2L in the metastatic setting. Of these patients, 36 had estimable TFI and 34 had started treatment no later than February 2022 allowing for a 6 month data accrual so were included in the TFI analysis of rwOS. Patients who had a TFI < 12 months (*n* = 18) or ≥ 12 months (*n* = 16) after (neo)adjuvant therapy had a similar median rwOS (11.1 [8.0–NE] and 10.9 [5.0–NE], respectively, Table 3).

## Discussion

This retrospective, observational cohort study investigated the characteristics of patients in the United States with mTNBC treated with SG in 2L and later line in the metastatic setting after its approval, as well as real-world SG use patterns, clinical outcomes, and tolerability. In this study, 2L and later-line SG provided a median rwOS of 10.0 months in all patients and 13.9 months in patients treated with SG in 2L in this broad, racially diverse patient population with poor prognostic factors. A similar benefit was observed for rwPFS and TTNTD. Outcomes from stratified analyses were generally similar to those observed in the overall population.

In the current study, the median age of patients was 60 years, and 40% of patients were 65 years and older; 17% had a poor performance status (defined as ECOG performance status ≥ 2). In addition, the population was also more racially diverse than is usually observed in clinical trials involving patients with BC [22]. In this study, 26% of patients were Black, a population often underrepresented in clinical trials, despite the fact that the incidence of TNBC is about twice as high for Black women (21%) than for White women (10%) in the United States [23–26]. In the ASCENT study, in patients with mTNBC, median age was 54 years, 80% of participants were White and 12% were Black, and the trial only enrolled patients with an ECOG performance status ≤ 1 [9]. There are a few reports in the published literature of SG use in real-world studies in patients with mTNBC. Data from “real-world” French, British, German, Italian, and United States studies have reported outcomes for patients treated with SG in 2L and later lines [15, 17–20]. Similar to the current study, patients in these real-world studies tended to have poorer performance status and a higher proportion of patients had visceral metastases and central nervous system disease compared with those in the clinical trials [15, 17–20]. In the current analysis, 111 patients were previously treated with a PD-(L)1 inhibitor in the metastatic setting while only 22 had PD-L1 positive results; this may be the result of missingness in the database (70% of patients had an unknown PD-L1 expression status); alternatively, patients may have been positive for microsatellite instability-high and tumor mutational burden-high as pembrolizumab is a preferred treatment for these patients [12].

After the initial accelerated approval of SG in the United States in April 2020 for patients with mTNBC who have received at least two prior therapies for metastatic disease, formal approval was extended to patients who have received at least two prior lines of systemic therapy, at least one in the metastatic setting (April 2021). Thus, the current study (spanning the April 2020 to May 2022 period) included institutions who were early adopters of SG. This may explain why most patients received SG as 3L + .

After the efficacy of SG was demonstrated in the controlled setting of the ASCENT clinical trial in patients with mTNBC, the current study demonstrates that SG also provides benefit in a broad population with poor prognostic factors in routine real-world clinical settings, including for patients receiving SG in 2L. Thus, treating patients in an earlier line in the metastatic setting improves clinical outcomes in this population of patients with a poor prognosis. In two other United States real-world studies of patients with mTNBC treated with chemotherapy in the 3L + setting, median rwOS ranged from 5.5 to 9.8 months, with eribulin providing the best outcomes [27, 28]. However, indirect comparisons with other real-world evidence studies are difficult as inclusion criteria, outcomes definitions, and censoring rules differ from study to study.

In the current study, toxicities were mainly managed with dose modifications and with supportive medication use such as G-CSF. Dose modifications (26% being dose reductions and 39% dose interruptions) rarely resulted in SG discontinuation (7%); these results are consistent with observations from the ASCENT study [9]. Concomitant administration of G-CSF was observed in 58% of all patients, the majority of whom had received G-CSF with prior anticancer treatment. The median time from the start of SG to administration of G-CSF was consistent with results from the ASCENT study [9]. SG treatment duration and maximum number of SG doses administered were higher among patients who received concomitant G-CSF treatment suggesting that longer treatment with SG can be achieved when neutropenia is properly managed.

The proportion of patients reporting fatigue was consistent with what was observed in the ASCENT study [9]; however, other rwAEs of interest (i.e., neutropenia; diarrhea, and alopecia) generally occurred in a lower proportion of patients in the current study, which likely reflect the known underreporting of rwAEs in some real-world studies, particularly retrospective studies. It is reasonable to assume that early-adopting institutions may have been involved in clinical trials that evaluated SG efficacy and safety; thus, improvement in knowledge and experience in managing rwAEs from these institutions may also have contributed to the lower proportion of reported rwAEs. Moreover, the low incidence of alopecia (11%) reported in patients treated with SG in this real-world setting might also reflect the fact that patients who experienced alopecia in earlier treatment lines (most had received prior chemotherapy) may have not reported it in later lines of therapy.

The main limitations of this study were those inherent to the collection of data from electronic health records, including missing or inaccurate data and underreporting of rwAEs, which might have affected clinical and tolerability outcomes. The regimen-based line of therapy algorithm used in this study was based on the availability and nature of the data within the ConcertAI Patient360™ database. As progression events may not be accurately reported in real-world data, a progression-based framework may not capture line of therapies adequately. Thus, the start of therapy and advancement to next line of therapy was defined based on a regimen-based framework, where a change of regimen, rather than disease progression, was considered advancement to the next line. The small number of patients in stratified analyses and the short follow-up period may also limit data interpretation. The data presented here included the first patients treated with SG in the United States after approval and, consequently, are more likely to be patients who had high disease burden and poor prognosis. Thus, these results may not be generalizable to the wider population of patients with mTNBC. Also, as data in the ConcertAI Patient360™ database focused on the United States population, results may not be generalizable to patients outside the United States.

In this real-world study, 2L and later-line treatment with SG was effective, irrespective of G-CSF use, race, and TFI, and demonstrated a tolerability profile consistent with findings from the phase III ASCENT study, despite the population in this real-world study being broader, more diverse, and with poorer prognostic factors than typically enrolled in clinical trials. The survival benefit and manageable safety profile in routine clinical settings show that SG is a valuable treatment option, as supported by international guidelines recommendations, and has potential to become standard of care for 2L mTNBC patients [12–14]. As real-world data with SG continue to accrue, they will provide further insight into the benefits of SG for this population of patients with limited therapeutic options.

## Supplementary Information

Below is the link to the electronic supplementary material.

Supplementary file1 (PDF 188 KB)

## Data Availability

Data to support the findings of this study were derived from the ConcertAI Patient360™ Breast Cancer dataset, but restrictions apply to the availability of these data, which were used under license for the current study and are thus not publicly available.

## Abbreviations

2L: Second-line
3L +: Third-line and later lines
AE: Adverse event
BC: Breast cancer
CI: Confidence interval
ECOG: Eastern Cooperative Oncology Group
G-CSF: Granulocyte-colony stimulating factor
HER2: Human epidermal growth factor receptor 2
HR: Hazard ratio
IHC: Immunohistochemistry
IQR: Interquartile range
mTNBC: Metastatic triple-negative breast cancer
NE: Not estimable
OS: Overall survival
PARPi: Poly(adenosine diphosphate-ribose) polymerase inhibitor
PD(L)-1: Programmed cell death protein death-(ligand) 1
PFS: Progression-free survival
rwAE: Real-world adverse event
rwOS: Real-world overall survival
rwPFS: Real-world progression-free survival
SG: Sacituzumab govitecan
TFI: Treatment-free interval
TNBC: Triple-negative breast cancer
TTNTD: Time-to-next-treatment or death
