# Supplementary material for: Real-world use patterns, effectiveness, and tolerability of sacituzumab govitecan for second-line and later-line treatment of metastatic triple-negative breast cancer in the United States
Source: Breast Cancer Res Treat. 2024 Jun 21;208(1):203–14. doi: 10.1007/s10549-024-07412-9 (PMC11452463; doi:10.1007/s10549-024-07412-9)

Supplementary Appendix

Real-world use patterns, effectiveness, and tolerability of sacituzumab govitecan for second-line and later line treatment of metastatic triple-negative breast cancer in the United States

Kevin Kalinsky, MD · Laura Spring, MD · Clinton Yam, MD · Manali Ajay Bhave, MD · Ioanna Ntalla, PhD · Catherine Lai, PharmD · Nikoleta Sjekloca, MD, PhD[4] · Brian Stwalley, PharmD, BCGP · Michael Stokes, MPH · Aliki Taylor, PhD · Rita Nanda, MD

Corresponding author:

Dr. Kevin Kalinsky

1365 Clifton Road Northeast, Bldg C, Fl 2

Atlanta, GA 30322

Phone: (404) 778-1900

Email: kevin.michael.kalinsky@emory.edu

ORCID number: 0000-0002-1583-3553

This appendix has been provided by the authors to give readers additional information about their work. Supplement to: Kalinsky K., et al. Real-world use patterns, effectiveness, and tolerability of sacituzumab govitecan for second-line and later-line treatment of metastatic triple-negative breast cancer in the United States.

Table of Contents

Supplementary Appendix 1

Supplementary Table 1 Baseline demographics and clinical characteristics of patients with mTNBC who received SG in 2L or 3L+ 2

Supplementary Fig. 1 Study design 5

Supplementary Table 1 Baseline demographics and clinical characteristics of patients with mTNBC who received SG in 2L or 3L+

**Characteristics**	**2L**<br>***n* = 77**	**3L+**<br>***n* = 153**
Age, median (IQR), y	59 (46–68)	60 (51–70)
< 65, No. (%)	48 (62)	91 (59)
≥ 65, No. (%)	29 (38)	62 (41)
Sex, No. (%)		
Female	77 (100)	153 (100)
Race, No. (%)		
White	48 (62)	98 (64)
Black	19 (25)	40 (26)
Asian	4 (5)	5 (3)
Other/Unknown	6 (8)	10 (7)
Ethnicity, No. (%)		
Hispanic or Latino	5 (6)	7 (5)
Other/Unknown	72 (94)	146 (95)
Treatment provider type, No. (%)		
Community	51 (66)	101 (66)
Academic	23 (30)	40 (26)
Unknown	3 (4)	12 (8)
Practice region, No. (%)		
Northeast	12 (16)	24 (16)
South	36 (47)	75 (49)
Midwest	17 (22)	33 (22)
West	12 (16)	21 (14)

**Characteristics**	**2L**<br>***n* = 77**	**3L+**<br>***n* = 153**
ECOG performance status,* No. (%)		
0 or 1	53 (69)	109 (71)
≥ 2	13 (17)	27 (18)
Unknown	11 (14)	17 (11)
Disease type, No. (%)		
Recurrent disease	62 (80)	108 (71)
De novo metastatic disease	9 (12)	32 (21)
Unknown	6 (8)	13 (8)
Time from mTNBC diagnosis to index date (or enrollment), median (IQR), months	6 (4.2–10.2)	15.5 (10.1–24.9)
Brain metastases at baseline, No (%)	8 (11)	9 (6)
Visceral metastases at baseline, No. (%)	60 (78)	107 (70)
PD-L1 expression status, No. (%)		
PD-L1 positive	8 (10)	14 (9)
PD-L1 negative	13 (17)	35 (23)
Unknown	56 (73)	104 (68)
*BRCA*1/2 mutation status, No. (%)		
Mutant	8 (10)	23 (15)
Wildtype	31 (40)	77 (50)
Unknown	38 (49)	53 (35)

2L second-line, *3L+* third-line and later line, *ECOG* Eastern Cooperative Oncology Group, *BRCA1/2* BReast CAncer gene 1/2, *ECOG* Eastern Cooperative Oncology Group, *IQR* interquartile range, *mTNBC* metastatic triple-negative breast cancer, *PD-L1* programmed cell death-ligand 1

*ECOG performance status records during the 6-month period before the index date until 2 months after the index date only were used. If ECOG performance status was not available during the 6-month period prior to the index date, Karnofsky scores were used instead (ECOG performance status 0 or 1 corresponds to Karnofsky scores 70–100 and ECOG performance status ≥ 2 to corresponds to Karnofsky scores ≤ 60)

†The proportion of patients may add up to greater than 100% as the subgroups are not mutually exclusive

Supplementary Fig. 1 Study design

BC breast cancer, *dx* diagnosis, *mBC* metastatic breast cancer, *SG* sacituzumab govitecan

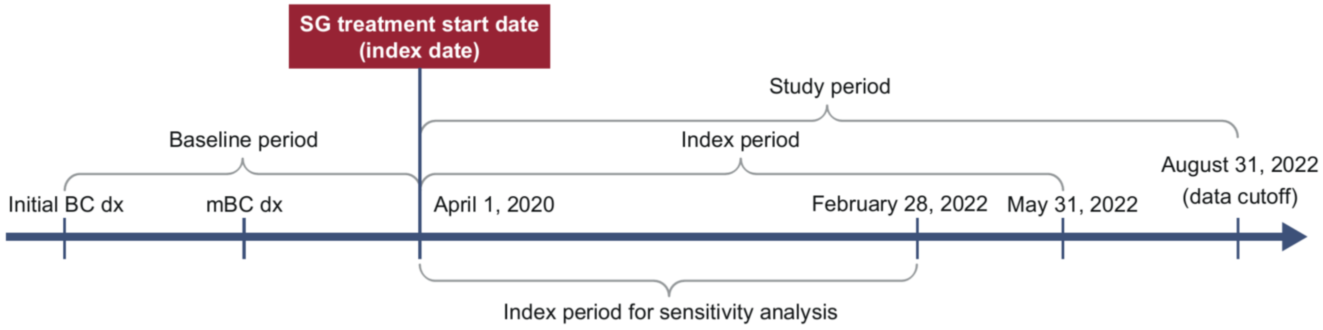

Supplement: Supplementary file 1 — Supplementary file1 (PDF 188 KB) [file 10549_2024_7412_MOESM1_ESM.pdf]
